# Oligomeric Nucleic Acids as Antivirals

**DOI:** 10.3390/molecules16021271

**Published:** 2011-01-28

**Authors:** Alessandra Mescalchin, Tobias Restle

**Affiliations:** Institute of Molecular Medicine, University of Lübeck, Center for Structural and Cell Biology in Medicine (CSCM), Ratzeburger Allee 160, D-23538 Lübeck, Germany

**Keywords:** therapeutics, viruses, inhibition, viral replication, oligonucleotides

## Abstract

Based on the natural functions and chemical characteristics of nucleic acids, a variety of novel synthetic drugs and tools to explore biological systems have become available in recent years. To date, a great number of antisense oligonucleotides, RNA interference-based tools, CpG-containing oligonucleotides, catalytic oligonucleotides, decoys and aptamers has been produced synthetically and applied successfully for understanding and manipulating biological processes and in clinical trials to treat a variety of diseases. Their versatility and potency make them equally suited candidates for fighting viral infections. Here, we describe the different types of nucleic acid-based antivirals, their mechanism of action, their advantages and limitations, and their future prospects.

## 1. Introduction

Generally, to prevent viral infection vaccines are used, which stimulate the immune system leading to an artificially acquired immunity against a specific virus. For this purpose, attenuated or inactivated viruses and in some cases viral structural proteins are applied in combination with adjuvants that enhance immune response. However, viruses such as hepatitis C virus (HCV) and human immunodeficiency virus (HIV) are able to evade the immune system, thus for those viruses no suited vaccine is available. Upon infection, viral replication can be kept under control by post-exposure drugs which can interfere with the viral entry into the host cell, the replication and assembly of viral components or the release of viral particles to infect other host cells. Many of the approved post-exposure antiviral drugs are small molecules, such as nucleoside analogues which act as competitive inhibitors, and are incorporated into the growing DNA chain by viral polymerases, instead of natural deoxynucleotide triphosphates (dNTPs). They lack a 3´-hydroxyl group on the ribose and thus prevent 3´-5´-phosphodiester bond formation, hence blocking further extension of the DNA [1,2]. One example is the HIV reverse transcriptase (RT) inhibitor 3´-azido-3´-deoxythymidine (AZT or zidovudine) [3] which was the first drug approved by the U.S. Food and Drug Administration (FDA) for the treatment of AIDS. AZT belongs to a class of nucleoside HIV RT inhibitors, which also includes the drugs didanosine, zalcitabine, stavudine, lamivudine, abacavir and emtricitabine. Further examples of nucleoside analogues are acyclovir used for the treatment of herpes simplex virus infections [4] and ribavirin for HCV [5]. The post-exposure antiviral drugs target different stages of the viral life cycle. Although treatment with these small molecules delay the progression of the disease, they do not cure it, mainly because drug-resistant mutants readily occur [6,7,8]. Further, complications arise from significant cytotoxic effects observed upon long term treatment with these antiviral therapeutics [9,10].

To overcome these obstacles and as an alternative to these antiviral therapeutics, oligomeric nucleic acid-based inhibitors have been developed to interfere with viral replication. They take advantage of the intrinsic properties of the virus and of its encoded information. Some of them interact with conserved regions within viral transcripts leading to a specific and efficient down-regulation of the target RNA. Others bind with high affinity to viral proteins inhibiting their activity. These oligonucleotides possess a number of advantages which make them interesting candidates for therapeutic applications, such as their high affinity and specificity towards a given target, the possibility to be selected against almost any molecule, the high inhibitory potential and the lack of toxicity and immunogenicity [11,12,13,14,15].

In the following sections we describe the different classes of oligomeric nucleic acids which have so far been produced and applied successfully as antiviral therapeutics over the last twenty years, their mode of action, and compare their advantages and limitations for treatment.

## 2. Antiviral Oligonucleotide-Based Therapeutics

**Figure 1:**
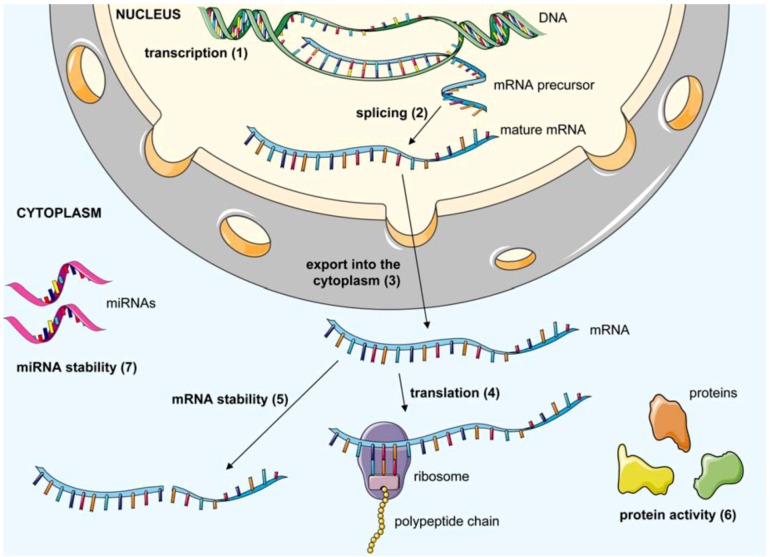
General overview of viral and cellular processes targeted by antiviral oligomeric nucleic acids. Transcription of viral genes (1) may be inhibited by decoys and aptamers which recognize viral regulatory elements or viral transcription factors. AsONs can be designed to bind exon-intron junctions blocking splicing (2) and maturation of mRNA precursors. The export of the viral mRNA into the cytoplasm (3) can be inhibited by asONs which bind the viral RNA or by decoys which bind to viral proteins involved in nuclear export of viral transcripts. Once the mRNA is in the cytoplasm, its translation (4) may be blocked by aptamers or asONs recognizing regions within or in the vicinity of the translation start site. AsONs, siRNAs, siDNAs and ribozymes can affect stability of the mRNA (5) by direct cleavage or by inducing its degradation via cellular enzymes. The activity of viral proteins (6) can be blocked by decoys, short oligonucleotides and aptamers which bind with high affinity to their targets. They can induce conformational changes of the protein or compete for binding with its natural substrate/ligand. In the specific case of HCV, a cellular miRNA (miR-122) has been shown to facilitate viral replication. Hence, miRNA inhibitors can be used to block and affect stability of specific miRNAs (7). This figure was produced using Servier Medical Art.

Oligomeric nucleic acid-based therapeutics can be subdivided into three main groups according to their target molecule. Antisense oligonucleotides (asONs), ribozymes, microRNAs (miRNAs) inhibitors (antagomirs), short interfering RNAs (siRNAs), and short interfering DNAs (siDNAs) belong to the first group. These oligonucleotides interact by Watson-Crick base pairing with viral or cellular transcripts leading to their degradation or functional blockage. The second group includes decoys, which are derived from nucleic acid sequences of protein ligands, and short oligonucleotides. Decoys antagonize natural ligands for their binding to particular proteins while short oligonucleotides are selected to interfere directly or indirectly with the function of a target protein. The third group includes aptamers with a defined tertiary structure that can be selected against highly structured viral nucleic acids as well as viral proteins. Here, we describe in detail the different groups of antiviral oligonucleotide-based therapeutics. Figure 1 summarizes the mode of action of these different inhibitors.

### 2.1. Classes of oligonucleotides targeting viral or cellular nucleic acids

#### 2.1.1. AsONs

In 1978, Zamecnik and Stephenson developed the first asON which was complementary to a 13 nucleotide (nt) sequence in the 3'- and 5'-long terminal repeats (LTRs) of Rous sarcoma virus RNA [16,17]. Treatment of infected cells with this asON led to a reduced virus production and in the wheat embryo cell-free system to inhibition of the translation of viral proteins. The asONs are usually synthetic single stranded DNA oligonucleotides, generally 12-30 nt in length, which interact via perfect Watson and Crick base-pairing with the target mRNA. 

There are several strategies by which asONs affect the expression of their target genes [18,19,20]. One of them is the activation of RNase H, a cellular enzyme which cleaves the RNA moiety in a DNA/RNA duplex, leading to degradation of the target mRNA. Another mechanism is the translational arrest of the target mRNA by steric blockade of the ribosome. This is achieved when the asON binds in the vicinity of the translational initiation site (AUG). Alternatively, binding of the asON either close to the cap or to the poly(A) tail of the mRNA may lead to destabilization and degradation of the transcript. AsONs can also be employed to inhibit mRNA maturation or to induce alternative splicing of the mRNA precursor. In this case, the asONs recognize and bind sequences spanning intron-exon junctions, and thus they sterically hinder the recognition and processing of those junctions by the spliceosome. 

Due to their length, RNA transcripts fold in very complex secondary and tertiary structures. Therefore, for the design and the selection of biologically active asONs, it is necessary to identify accessible target sites on the target RNA. For this purpose, theoretical and experimental approaches are generally used [21,22,23]. The theoretical methods are based on *in silico* RNA structure models by using algorithms which predict structurally favourable sites for asON targeting [24,25]. This approach is typically fast, cheap and can be applied for high throughput screening, though in same cases, the predicted structures might not represent the real folding of the RNA molecule in solution. On the contrary, experimental approaches are more time-consuming and expensive, but rely on real RNA structures and real annealing in solution. An example is the ´gene-walking strategy´ in which asONs spanning the entire target sequence are tested *in vitro* or *in vivo* for efficient down-regulation of the target gene. An even more sophisticated approach makes use of oligonucleotide-based arrays to map the hybridization sites on the RNA molecule. Alternatively, the selection of asONs which can induce RNase H-mediated cleavage of mRNA can be performed by incubating a library of asONs with the labelled target RNA and the RNase H enzyme. Accessible sites and active asONs can be revealed by evaluation of the extent of cleavage induced by individual asONs.

Based on their versatility and ability to specifically down-regulate their target transcripts, asONs are applied and tested for the treatment of a variety of diseases including viral infections, cancer, inflammation and cardiovascular diseases [26,27,28,29]. So far, only one asON received FDA approval, *i.e.,* Fomivirsen (Vitravene^TM^) developed by Isis Pharmaceuticals. This 21 nt phosphorothioate (PS)-modified asON inhibits the expression of viral proteins from the major immediate-early transcriptional unit of cytomegalovirus (CMV). Hence, it is used for the treatment of HIV-associated CMV retinitis infections and administered by repeated injections into the eye [30,31]. An alternative antisense-based antiviral approach relies on a 937 nt RNA molecule complementary to the *env* gene of HIV [32]. This antisense RNA, called VRX496, is inserted into a HIV-1-based lentiviral vector which retains the long LTRs of HIV, thus its expression is up-regulated upon infection with wild type HIV. When the virus starts to replicate in the host cell, the antisense RNA prevents the production of the envelope protein, thereby blocking the HIV replication cycle. VRX496 is currently being tested in phase II clinical trials. Another promising approach to inhibit HIV was developed by Matzen *et al.* [33] which is based on an asON complementary to the polypurine tract. The viral RNase H, activated by this asON/viral RNA heteroduplex, cleaves the viral genome and thus destroys it. In the case of positive-strand RNA viruses, such as HCV, the replication takes place entirely in the cytoplasm of the host cell. Therefore, for those viruses an asON-mediated cleavage of the viral RNA by the nuclear enzyme RNase H is not possible. ISIS developed a 20 nt PS-modified asON (ISIS-14803) which targets a region within the internal ribosome entry site (IRES) of HCV RNA and acts via a steric block mechanism [34]. ISIS-14803 was tested in clinical trials, but it showed a very low anti-HCV activity in patients, thus studies were discontinued [34]. Recently, Warren *et al.* [35,36] described the application of positively charged phosphorodiamidate morpholino-modified asONs in nonhuman primates infected with Zaire Ebola virus (ZEBOV) or Lake Victoria Marburg virus (MARV). The recovery rate of infected monkeys upon treatment with asONs was 60% and 100% after infection with ZEBOV and MARV, respectively. These asONs, which will soon be entering phase I clinical trials, sterically hinder the translation of VP24 and VP35 viral transcripts which are important for ZEBOV and MARV replication. 

#### 2.1.2. Ribozymes

Ribozymes are single stranded RNA molecules (50-100 nt) having a well defined tertiary structure allowing them to catalyze chemical reactions without the requirement of any protein [37,38,39]. They are naturally occurring molecules originally discovered in *Tetrahymena thermophilia* by T.R. Cech who was awarded in 1989 the Nobel Prize for Chemistry [40]. Ribozymes catalyze reactions such as hydrolysis of phosphodiester bonds, peptide bond formation (ribosomal RNA), ligation, as well as polymerization [41,42] and can be subdivided into *cis*- (intramolecular catalysis) or *trans*-acting (intermolecular catalysis) molecules. 

**Figure 2:**
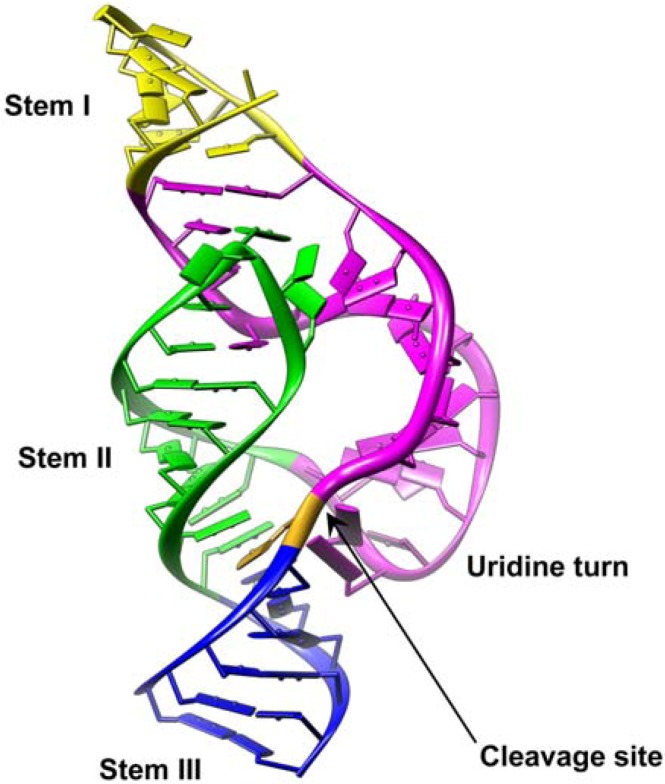
X-ray structure of full-length *Schistosoma mansoni* hammerhead ribozyme. The ribozyme is coloured according to secondary structure elements and domains (PDB: 2GOZ). The arrow points to the scissile bond.

Generally, reactions mediated by ribozymes are multiple-turnover and sequence-specific, both characteristics make these molecules suited candidates for therapeutic application. For this purpose, usually *trans*-cleaving hammerhead ribozymes are used, which are characterized by a central catalytic domain and two flanking arms (6-12 nt) complementary to the target RNA (Figure 2). The cleavage site of the hammerhead ribozyme on the target RNA is downstream the H nucleotide of a NUH triplet (N = any nt; H = A, C or U) [43]. To improve stability and affinity for the target, modified nucleotides such as locked nucleic acids (LNA) or 2´-O-methyl (2´-OMe), which are briefly described in section 4, are commonly introduced into the flanking arms of the ribozyme [44]. Ribozymes can be endogenously transcribed from vectors or chemically synthesized and delivered to cells [45].

Several clinical studies have been performed in the use of ribozymes to treat infectious diseases, in particular HCV and HIV infections. The genome of HCV is a positive-strand RNA containing several NUH motifs [46,47]. A ribozyme, called Heptazyme targeting the highly conserved IRES in the 5´-untranslated region (5´-UTR) of the HCV genome, was tested in Phase II clinical trials. Heptazyme led to a 10% reduction of the viral load in the serum of patients. However, toxicological tests for this ribozyme were not encouraging and led to discontinuation of the clinical studies [43,48]. Most recently, Lévesque *et al.* [49] coupled anti-HCV ribozymes with a specific on/off adaptor, called SOFA, which enable cleavage activity only in the presence of the desired RNA substrate increasing the selectivity of the ribozymes. They examined *in vitro* the ability of SOFA-coupled ribozymes to inhibit HCV replication using a luciferase-based replicon and observed up to 40% reduction in the luciferase activity. 

For the retrovirus HIV-1, a *tat*-*vpr*-specific anti-HIV ribozyme, called OZ1, is currently been tested in phase II clinical trials [50]. OZ1 consists of a Moloney murine leukemia virus-based gammaretroviral vector (LNL6) containing a gene that encodes a ribozyme targeting the overlapping *vpr* and *tat* reading frames of HIV-1. This approach is based on a gene transfer by which the DNA of the OZ1 retroviral vector is integrated into hematopoietic stem cells. *In vitro* experiments showed long-term inhibition of HIV-1 replication by OZ1 in cell culture without occurrence of escape mutants. In HIV-infected patients treated with OZ1, the CD4^+^ lymphocyte counts were higher than in the group treated with placebo. Then again, over time a decline in the percentage of patients who maintained the integrated DNA from the OZ1 retroviral vector was observed [50,51]. 

Recently, a very promising hammerhead ribozyme (HHPol13) targeting the HIV-1 *pol* gene was also described [52]. The antiviral effect of HHPol13 was compared to the one of an siRNA targeting the same region in the viral *pol* gene. Results showed that the ribozyme led to a drastic reduction of HIV-1 replication in cell culture experiments while the siRNA showed no inhibition.

#### 2.1.3. MiRNAs and their inhibitors

MiRNAs are short non-coding RNAs that can be derived from cellular or viral transcripts [53,54]. More than 700 different miRNAs have so far been identified in humans, where they participate in the post-transcriptional regulation of gene expression. Most of them are transcribed by the RNA polymerase II as primary RNA precursors which are several hundred nt in length and undergo processing by the cellular RNase III enzyme Drosha into pre-miRNAs (~60 nt). The pre-miRNAs are then exported from the nucleus to the cytoplasm by Exportin-5 and further processed to generate double stranded miRNA intermediates [55]. These miRNA intermediates are loaded into the RNA-induced silencing complex (RISC) where a strand selection takes place and the strand with the lower thermodynamic stability at its 5´-end, called guide strand, is incorporated to form miRISC [56,57]. The other strand, called passenger strand, is normally degraded. In some cases, both strands of the miRNA intermediate are viable and can form active miRISCs [58]. The loaded strand acts as a guide to direct the miRISC to complementary mRNA species. Depending on the level of complementarity between the miRNA and the target region on the mRNA, the miRISC can either cleave the mRNA or inhibit its translation. Generally, the cleavage of the target mRNA takes place when miRNA and target mRNA are fully complementary, partial complementarity (between the 2^nd^ and the 7^th^-8^th^ nt from the 5´-end of the guide strand) instead leads to repression of mRNA translation [59,60]. Typically, one miRNA can inhibit the translation of up to hundreds of different mRNAs and there are several possible mechanisms by which this may occur, such as promoting ribosome drop-off, degradation of mRNA, interfering with initiation of the translation via inhibiting the circularization of the mRNA or competing for binding to the cap [59,61].

MiRNAs have been described to be involved in the development of a variety of diseases such as cancer, asthma, heart diseases and allergies [62,63,64,65]. Based on their weak target specificity and on their participation in the development of diseases, usually cellular miRNAs are considered targets for therapy rather than drugs. Hence, in the last years, miRNA inhibitors, so-called anti-miRs, anti-miRNAs or antagomirs, have been developed which are chemically modified, single-stranded oligonucleotides fully complementary to the guide strand of the target miRNA [66,67,68,69]. They are designed to specifically bind endogenous miRNAs, blocking their function. The miRNA inhibitors are chemically modified with the dual purpose to stabilize them and to improve their affinity for their targets. The chemical modifications commonly used for the miRNA inhibitors are the 2´-OMe and LNAs (see section 4). The first modification provides higher nuclease resistance and binding affinity toward RNA compared to unmodified oligonucleotides, the LNA modification provides very strong duplex formation with the target RNA and displays excellent mismatch discrimination, reducing off-target effects [69,70,71].

Interestingly, some viruses were also shown to encode miRNAs, in particular the nuclear DNA viruses, such as herpesviruses, polyomaviruses and adenoviruses [54,72]. Recent data suggest that viral miRNAs may play important roles in viral replication by down-regulating host cell factors involved in cell survival and in antiviral immune response [73] or by inhibiting the expression of virally-encoded proteins, e.g., early regulatory proteins, and therefore stabilizing the viral latent state [74]. An example of a host target protein is the natural killer cell ligand MICB, which is involved in mediating immune responses and is down-regulated by miRNAs encoded by the human CMV, the Kaposi’s sarcoma-associated herpesvirus, and the Epstein-Barr virus [75,76]. These miRNAs contribute to the viral pathogenic and replication properties, thus blocking their action by miRNA inhibitors may represent a strategy to fight viral infections [77]. In addition, most of the identified viral miRNAs have only little homology to host cell miRNAs [78], thus leading to a reduced off-target effect by the miRNA inhibitors and to an increased therapeutic potential.

However, viruses are not only able to encode their own miRNAs, but also to exploit cellular miRNAs for their own purposes [76,79]. An example is HCV which uses a highly conserved liver-specific miRNA, termed miR-122, to facilitate its replication and translation, but the regulatory mechanism by which this is achieved remains unclear [79,80,81]. HCV is a single stranded positive-sense RNA flavivirus, and its genome contains two miR-122 binding sites in the 5´-UTR. Interaction of miR-122 with the HCV genome has been shown to increase accumulation of viral RNA in cultured liver cells, modulating HCV abundance [80,82]. Therefore, down-regulation of miR-122 via a complementary inhibitor seemed a potential strategy for HCV treatment. Initial experiments were performed in Huh7 cells using a 2´-OMe RNA oligonucleotide fully complementary to miR-122 to determine the relationship between the miRNA and the viral replication [80]. Krützfeldt *et al.* developed a miR-122 inhibitor with 2´-OMe and phophorothioate (PS) chemistries and a cholesterol group at the 3´-end which facilitated its application *in vivo* [67]. Injections of this inhibitor into the tail veins of mice led to an efficient and specific suppression of endogenous miR-122. *In vivo* studies performed by Santaris Pharma showed that LNA modifications are also suited to silence miR-122 in mice and primates [83,84]. A miR-122 inhibitor (SPC3649) was developed with an optimized combination of LNA and DNA bases and a PS backbone [84] which led to specific and stabile down-regulation of the target and to low toxicity. To assess its therapeutic potential, SPC3649 was tested in chimpanzees chronically infected with HCV [85]. Results showed that the treatment with SPC3649 led to reduction of viral load by up to 2.6-fold for HCV genome equivalents in serum and to an improved liver histology. SPC3649 has entered clinical trials in September 2009 and has a strong potential as a therapeutic agent. 

#### 2.1.4. SiRNAs and siDNAs

SiRNAs are 21 to 22 nt double stranded RNAs involved in the RNA interference (RNAi) pathway by which gene expression is down-regulated at the post-transcriptional level [86]. SiRNAs induce gene silencing via mediating sequence-specific cleavage of perfectly complementary mRNA. They can be synthetically produced and delivered to cells [87] or endogenously transcribed from vectors [88,89,90] as two single stranded RNAs, or as short hairpin RNAs (shRNAs) that are processed into siRNAs by the cellular Dicer enzyme. The siRNAs are then incorporated into the RISC which contains Argonaute 2 (Ago2) protein. One of the two siRNA strands, called antisense or guide, is perfectly complementary to the target mRNA while the other, called sense or passenger, is cleaved, and thus eliminated by Ago2 from the complex [56,57]. The active RISC containing the guide strand and Ago2 recognizes the target mRNA and cleaves it.

The first evidence of their therapeutic potential *in vivo* was provided by Song *et al.* [91]. In this study, mice with autoimmune hepatitis were treated with siRNAs targeting the gene *Fas* which is involved in programmed cell death [91]. Results showed that silencing *Fas* expression by siRNAs protected the mice from liver cytotoxicity and fibrosis. Since then, much progress has been made concerning the design and delivery of siRNAs improving particular features for the *in vivo* application [92]. 

For the design of siRNAs to achieve maximal mRNA knockdown along with a lack of undesired side effects, several parameters need to be considered, such as the folding of the target mRNA [93,94], its association with proteins which may interfere with RISC binding, and off-target effects [95]. There are several methods for siRNA design which can be subdivided into theoretical and experimental approaches. The theoretical methods include the rational design that is based on algorithms and can be performed by using available online tools [96,97], and the conventional method which select siRNAs based on particular criteria, e.g., GC content, location of the seed region within the target RNA and stability of the ends. However, the efficacy of the designed siRNA molecules still needs to be determined empirically. Efficient siRNAs can also be screened experimentally, *i.e.* by using RNAi microarrays or comparing *in vitro* or *in vivo* the gene-silencing ability of libraries or a set of lead candidate siRNA sequences [98].

To date, numerous studies have been conducted in the use of siRNAs as antivirals. The best suited viruses for RNAi-based therapeutics are the respiratory viruses since lungs, as target tissues, are relatively easy to reach [99]. An example is the respiratory syncytial virus (RSV) which is a negative-strand RNA virus. In mice, replication of RSV was successfully blocked by siRNAs [100]. Alnylam developed an siRNA (ALN-RSV01) directed against the viral transcript encoding the N-protein of RSV. This siRNA was found to be safe and well tolerated when administered intranasally [101]. ALN-RSV01 is now in phase II clinical trials.

A major limitation in antiviral therapy is the emergence of viral escape mutants that are no longer targeted by a given drug. In case of siRNA therapeutics escape mutants hamper the recognition of the target sequence by the RISC. Mutations were found within the siRNA target sequence [102] or in its vicinity, thus causing a conformational change of the RNA target structure [103]. To overcome this problem, new combinatorial approaches have been developed in which multiple siRNAs targeting different viral genes are applied simultaneously [104,105]. Tekmira Pharmaceuticals indeed developed an RNAi-based therapeutic approach for protecting nonhuman primates from ZEBOV, which consists of a combination of modified siRNAs targeting the viral L polymerase, viral VP24, and VP35, respectively [105]. The siRNAs were encapsulated in uniform lipid nanoparticles, termed stable nucleic acid-lipid particles (SNALPs) [106,107] that facilitate cellular uptake of the siRNAs and their endosomal release. All primates treated with these modified siRNAs survived and they were free from ZEBOV two weeks after infection. This anti-Ebola virus approach is currently in pre-clinical trials.

Recently, Nowak *et al.* [108] described the phenomenon of DNA interference (DNAi) in which siDNAs are capable of sequence-specific inhibition of gene expression such as their RNA counterpart. They evaluated the effect of a HIV-1 gp41-directed siDNA in HeLa cells expressing a gp41-EGFP fusion protein, and they observed a significant decrease in the amount of gp41 expression, both at the protein as well as at the transcript level.

### 2.2. Classes of oligonucleotides targeting viral proteins

#### 2.2.1. Decoys

Decoys are usually single- or double-stranded oligonucleotides (30-100 nt in length) which fold in complex three-dimensional (3D) structures and shapes. They are designed according to the nucleic acid consensus sequence recognized by a particular protein, therefore they possess a structure which mimics or is identical to the real ligand, thus acting as ligand antagonists [45].

Decoys are widely used for target validation and for therapeutic application [11] and show high affinity toward their target very much like aptamers (see below). The first decoy was described for the treatment of HIV-1 infection and consisted of a transactivation response element (TAR) which was used to render cells resistant to HIV-1 replication [109]. The Tat protein is required for the transcription of the viral genome and binds to the viral TAR RNA during transcription. The TAR decoy mimics the viral TAR RNA hairpin structure and competes with it for the binding to Tat. Further studies demonstrated that a single nucleotide change which disrupts the stem structure of TAR abolishes the ability of the TAR decoy to inhibit HIV-1 replication, while a compensatory mutation which restores the stem structure also restores the functionality of the TAR decoy [110]. Bohjanen *et al.* [111] developed a circular TAR decoy which showed an exceptionally high stability in HeLa nuclear extracts, when compared to the classical linear TAR decoy. Moreover, the circular TAR decoy was able to efficiently inhibit Tat *in vitro* in its function of trans-activating transcription from the HIV-1 promoter. More recently, Anderson *et al.* [112,113] combined multiple highly potent anti-HIV-1 transgenes in a single gene therapy vector to treat HIV-1 infection. The use of a single anti-HIV drug would not be sufficient for long-term antiviral protection because of raise of viral escape mutants. Therefore, combining different drugs in a unique vector may avoid development of resistant strains. This was achieved by combining a TAR decoy, a shRNA targeting *rev* and *tat*, and a CCR5 ribozyme into a lentiviral vector. This construct, called Triple-R, was tested using the severe combined immunodeficiency (SCID)-hu mouse human thymopoiesis model. The CD34^+^ hematopoietic stem cells transduced with Triple-R developed normally into HIV-resistant T cells *in vivo* [112]. 

Another RNA decoy to inhibit HIV-1 infection is the rev responsive element (RRE) decoy [114]. The viral protein Rev recognizes RRE on the unspliced viral transcript and transports it from the nucleus into the cytoplasm of the host cell. When the RRE decoy was expressed in infected cells, it sequestered the Rev protein blocking the viral replication cycle. In a phase I clinical trial CD34^+^ cells were transduced *ex-vivo* using a retroviral vector carrying this RRE decoy and reinfused into patients with no side effects [115]. However, analysis of venous blood samples one year after gene transfer revealed very low levels of cells carrying the vector, thus the study was discontinued.

#### 2.2.2. Short oligonucleotides

Short oligonucleotides, below 12 nt in length, have been shown to specifically interact with target proteins [116,117,118]. Due to their small size, these short nucleic acids lack the disadvantage of a costly chemical synthesis, which is the main limiting factor in the use of longer oligonucleotides, while preserving advantages, such as the possibility of screening libraries systematically to identify binders, and of a rational drug design. 

Pinskaya *et al.* [117] showed binding of a PS-modified octanucleotide to HIV-1 integrase (IN). This oligonucleotide was originally derived from the LTRs of the virus genome. The binding to IN was sequence specific, even though the high affinity was due to the PS-modified backbone of the oligonucleotide. Likewise, Wyatt *et al.* [116] showed that an 11mer oligonucleotide, which was coupled with an acridine group, bound with high specificity to the surface glycoprotein 120 (gp120) of HIV-1 and was able to inhibit gp120 mediated cell fusion. Albeit, despite of a sequence specific interaction the high affinity was primarily caused by the hydrophobic acridine moiety, forming additional contacts between the enzyme and the oligonucleotide. 

Another study [118] described the specific interaction between a hexanucleotide (Hex-S3) and the HIV-1 RT. Hex-S3 interacted with HIV-1 RT in a highly sequence specific manner and did not bind to the closely related enzymes of HIV-2 and EIAV (equine infectious anemia virus). Binding studies using a set of Hex-S3 derivatives indicated that the specific interaction depended both on the length and sequence of the oligonucleotides with a *K*_d_ of ~ 5.3 µM. In human cells, Hex-S3 proved to suppress HIV-1 viral particle production by up to three orders of magnitude in a dose-dependent manner (IC_50_ ~ 1.8 µM), indicating that it exerted specific and biologically relevant activity. 

Generally, to select for protein ligands combinatorial approaches are performed in solution. However, in the specific case of nucleic acids these methods involve cloning steps for sequencing that potentially might lead to the introduction of a bias for certain sequences, which are preferentially accepted by the cloning enzymes. Additionally, an erroneous amplification might occur, leading to wrong sequence information. Therefore, to overcome these limitations, a hexanucleotide-based array was developed [119] in which the complete sequence space of hexamers (4^6^ = 4,096) was immobilized to a solid support. Analyses with different target proteins clearly showed that this approach is suited for the identification of novel drug candidates.

### 2.3. Classes of oligonucleotides targeting viral nucleic acids or viral proteins

#### 2.3.1. Aptamers

Aptamers (from the Latin *aptus* = to fit) are 30 to 100 nt long single stranded DNA or RNA oligonucleotides which fold into complex, 3D shapes rivaling those of the target molecules. They interact with high specificity and affinity with their target molecule and the tight binding often affects the activity of the target molecule [120,121]. These synthetic high-affinity nucleic acids ligands are selected from a large pool of randomised sequences by a process called systematic evolution of ligands by exponential enrichment (SELEX) which allows the screening of aptamers for binding to target molecules [122,123,124]. So far, this approach has allowed the selection of high-affinity DNA or RNA aptamers for a variety of targets including small molecules such as amino acids and antibiotics, proteins and even complex targets, *i.e.* whole cells and viruses [125,126,127,128,129]. 

Aptamers have a very broad application range, and are used as diagnostic agents, for target validation and clinical application [12,130,131,132,133,134]. Additionally, due to their high similarity in function and affinity towards the targets, aptamers are used as substitutes for antibodies [121,135] and applied in several systems as capture agents, to monitor protein-nucleic acids and protein-protein interactions [136]. Further, a high-affinity ligand selected against a plasma membrane receptor, the prostate-specific membrane antigen receptor, has been coupled to siRNAs and used to mediate siRNA cell type-specific delivery [137]. These molecules are well suited for therapeutic applications because of their remarkably high affinity and specificity towards their targets, the lack of toxicity and immunogenicity [11,12].

For therapeutic purposes, generally viral or cellular proteins or nucleic acids are used as target molecules for the selection of aptamers. In 2004 the first aptamer received FDA approval. This RNA aptamer, called Pegaptanib sodium or Macugen, was developed by Eyetech/Pfizer´s against the vascular endothelial growth factor (VEGF) [138]. Binding of Macugen to its target protein, prevents choroidal neovascularization and thus, it is applied for the treatment of age-related macular degeneration. Up to now, many aptamers have been selected with the goal to develop novel antiviral agents [13]. Generally, antiviral aptamers can be subdivided into two main groups, *i.e.* aptamers targeting viral nucleic acids and aptamers targeting viral or cellular proteins. 

The first group includes RNA as well as DNA aptamers. Recently, Srisawat and Engelke selected RNA aptamers which bind with high affinity to the LTRs of HIV-1 DNA and can be used as gene-directed therapeutic agents [139]. Most likely these aptamers interact via Watson-Crick base-pairing with one strand of the DNA forming stable complexes. Another HIV-1 target is the TAR element RNA which is an RNA hairpin located in the 5´-UTR of HIV-1 mRNA and it is essential for viral replication. To date, several aptamers that generate highly stable and specific complexes with TAR have been identified [140,141,142,143,144]. For HCV generally the IRES of the viral mRNA where translation begins is used for the selection of aptamers. RNA aptamers which specifically bind to domain II and domain III-IV of the HCV IRES, respectively, and inhibit IRES-dependent translation have been isolated [145,146]. The minus-strand of the IRES which corresponds to the 3´-terminal end of the minus-strand HCV RNA was also successfully used as target for aptamer selection. This region is highly conserved among HCV subtypes and is essential for HCV replication since the viral RNA-dependent RNA polymerase (RdRp) recognizes it as the initiation site for plus-strand synthesis of the HCV genome. An identified RNA aptamer inhibited plus-strand synthesis *in vitro* of about 2-fold [147]. 

**Figure 3:**
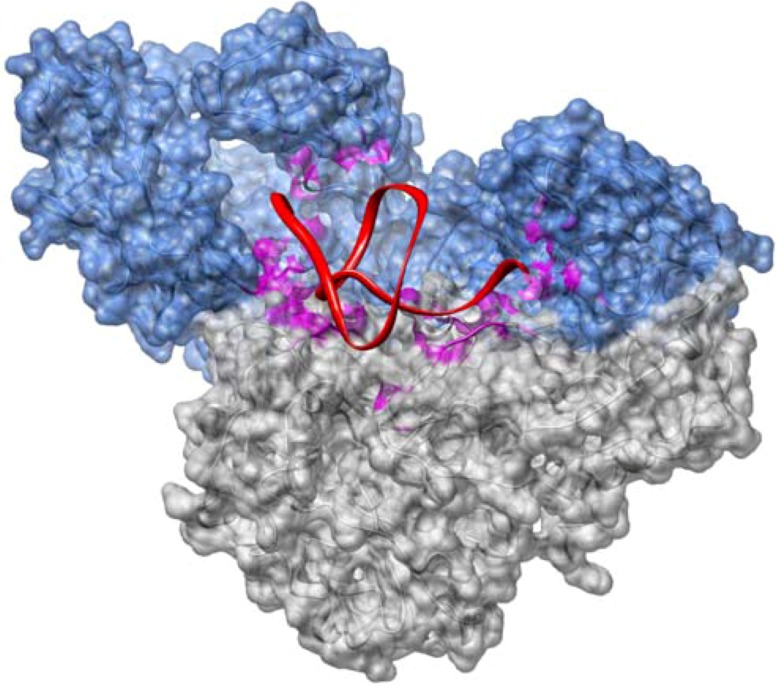
Structure of the HIV-1 RT heterodimer complexed with a 33 nt pseudoknot RNA aptamer (PDB file: 1hvu). The color coding is as follows: p66 (blue), p51 (grey) and RNA (red). Surface residues < 5 Å away from the pseudoknot inhibitor are colored in magenta.

The second group includes aptamers targeting viral proteins which play a key role in viral infection and replication. For example, for the treatment of HIV-1 infection, potent aptamers [148,149,150,151,152,153,154,155] were selected against RT. This retroviral enzyme represents an excellent target because of its key role in the viral replication cycle, and a missing ortholog in mammalian cells [156]. A particularly interesting example is an aptamer originally identified by Tuerk *et al.* [157]. This RNA aptamer shows a typical pseudoknot fold (Figure 3). Further detailed structural and biochemical studies by others revealed an astoundingly strong binding to the target with an affinity < 25 pM and a strong inhibitory effect on viral replication [158,159,160]. Most of the selected aptamers prove to interfere with more than one step during the reverse transcription process [161]. Hannoush *et al.* [152] selected aptamers that proved to inhibit the RNase H activity of RT without a measurable inhibitory effect on polymerase activity by directly screening a limited library of chemically synthesised RNAs designed to adopt hairpin and dumb-bell configurations. In addition to RT other HIV-1-encoded proteins such as the regulator of virion expression (Rev), the IN, the transactivator (Tat), the glycoprotein 120 (gp120) and the nucleocapside were successfully employed for selection of high-affinity ligands [13,121]. 

To increase target-specificity and avoid the occurrence of escape mutants, bivalent aptamers [162] could be used which consist of two aptamer domains assembled through a central stem structure. This would allow simultaneous recognition and binding to two different regions on the target molecule increasing binding specificity and reducing the incidence of viral escape mutants.

To date, many modifications of the SELEX process have been developed to improve specificity, affinity and stability of the selected aptamers. For example, the negative-SELEX is based on a pre-selection of nucleic acid ligands to minimize the co-selection of unwanted binders. This is achieved by incubation of the initial pool of DNA or RNA sequences with one or more protein/s which should not be recognized by the aptamer [120]. The Toggle-SELEX allows isolation of aptamers with a broader range of specificities by selecting against related targets in alternating cycles [163]. Moreover, the availability of less stringent variants of RNA and DNA polymerases [164,165,166] has allowed introduction of modified nucleotides into the sequence of aptamers, increasing their stability in physiological fluids [167]. For example, the double mutant Y639F/H784A T7 RNA polymerase can incorporate nucleotides with bulky groups at the 2´-position including 2´-azido and 2´-OMe pyrimidines. Likewise, the stoffel fragment of *Taq* DNA polymerase incorporates 2´-OMe nucleotides. Nowadays, the SELEX procedure is frequently performed as an automated *in vitro* process allowing the high-throughput selection against almost any target [168,169].

The SELEX process has been also adapted to produce high-affinity molecules called Spiegelmers (from German *Spiegel* = mirror) [170]. These molecules developed by the company NOXXON are derived from L-oligonucleotides which are resistant to the activity of nucleases. Hence, they are stable in human plasma for at least 60 h at 37 °C while unmodified RNA or DNA counterparts typically are degraded in seconds to minutes. Spiegelmers are interesting candidates for a therapeutic application since they show similar binding affinities as compared to DNA or RNA aptamers and antibodies while being essentially inert with respect to enzymatic degradation.

## 3. Advantages of Oligonucleotide-Based Drugs

A major advantage of oligonucleotide-based drugs compared to conventional small molecule, peptide- or protein-based drugs is the fact that in most instances they can either be easily designed obeying simple Watson-Crick base pairing rules or isolated from libraries employing straight forward selection protocols like SELEX. Moreover, since they represent simple polymers of either ribo- or deoxynucleotide monomers their physicochemical and pharmaceutical properties are comparable regardless of size and sequence composition. Thus, a given application scheme might be applicable for many different nucleic acid-based drugs. In general, oligonucleotides show a binding affinity in the pico- to low nanomolar range and are highly specific for their desired targets. For molecules which exert their inhibitory effect via Watson-Crick base pairing, one or two mismatches over a stretch of approximately 20 nt are typically sufficient to render them inactive or at least drastically reduce their activity. By the same token, there are examples of aptamers differing by 3-4 orders of magnitude in binding affinity for even highly related targets. Furthermore, compared to peptide- or protein-based drugs nucleic acids are virtually non-immunogenic. 

## 4. Limitations of Oligonucleotide-Based Drugs

Despite several advantages of oligonucleotide-based inhibitors over traditional drugs, there are certain limitations which need to be considered. In general, the stability of nucleic acids is rather limited with serum half-times in the range of seconds to minutes. To overcome this problem a variety of chemical modifications of either the base, ribose or phosphate backbone of the polymers are available. Common alterations are for example PS and 2´-O-alkyl modifications (*i.e.*, 2´-OMe). More recently peptide nucleic acids (PNAs) or LNAs are used among others, each of which showing advantages and disadvantages for a given application [18]. Another problem of nucleic acid-based drugs is their fast renal clearance rate with half-times in the range of minutes. Again this obstacle can be relatively easy overcome enlarging the molecular weight of the polymers by complexation for example with liposomes or by site-specific addition of polyethylene glycol (PEG), a procedure referred to as PEGylation [171]. Taken together, such modifications bring up serum half-times and renal clearance rates in the order of days.

**Figure 4:**
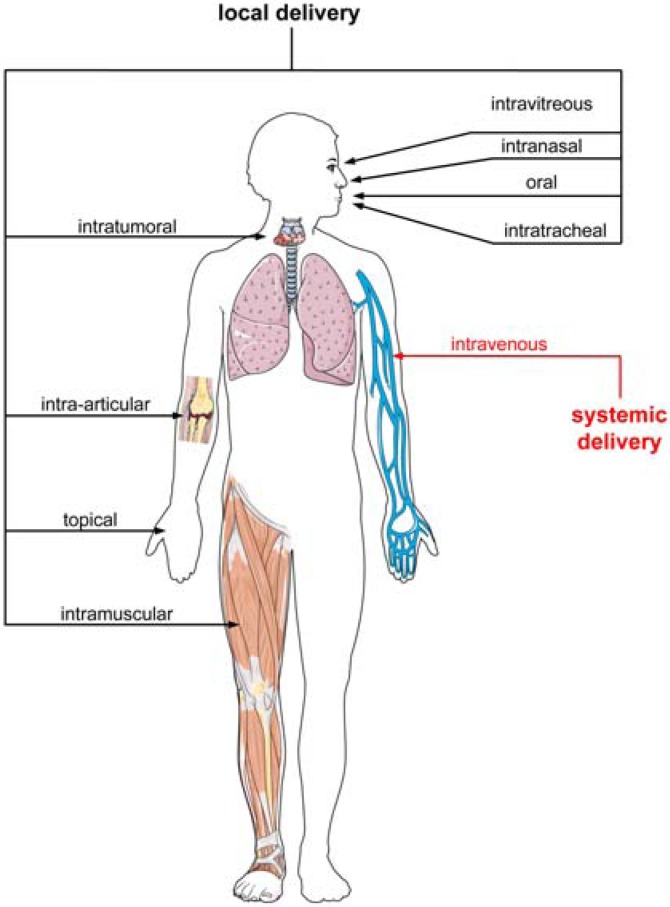
Possible routes for *in vivo* nucleic acid administration. Shown are local and systemic administration schemes. This figure was produced using Servier Medical Art.

While stability and clearance issues are manageable, which also holds true for so called ´off target effects´, potential activation of the innate immune system or undesired side effects caused by saturation of endogenous pathways in the case of shRNAs [172], there remains the essentially unresolved and highly challenging problem of nucleic acid delivery. As outlined in Figure 1, most of the targets of potential oligonucleotide-based therapeutics are localized inside the cell. Thus, as a prerequisite for efficacy they need to cross the membrane barrier. Beside viral vectors there are a variety of non-viral systems like cationic polymers, cationic liposomes, polymeric nanoparticles and cell-penetrating peptides which represent attractive concepts to bypass the problem of poor membrane permeability of these charged macromolecules. The different approaches have been described in recent reviews [60,173,174]. Possible routes for *in vivo* nucleic acid delivery are depicted in Figure 4. In some cases there is a flowing transition between local and systemic delivery; *i.e.,* intranasal, intratracheal or topical. The oligomeric nucleic acids can be applied unmodified or chemically modified either naked or in combination with a carrier. Alternatively, entire plasmids or viral vectors encoding the desired sequence can be injected leading to a transient or stable endogenous expression of the corresponding oligonucleotide.

## 5. Conclusions and Perspectives

While most of the oligomeric nucleic acids described above have evidently been shown to be potent inhibitors of viral replication *in vitro* their clinical use is still rather limited owing to the fact that appropriate delivery systems are missing. In this context it is not a surprise that only a single compound Vitravene^TM^ has so far been approved by the FDA. Thus, the development of effective and safe delivery systems for therapeutic oligonucleotides is of utmost importance. Besides viral vectors there is a highly diverse and constantly increasing number of non-viral systems evolving. Yet, at present even the most advanced systems either lack the efficiencies required for downstream drug development or do show a substantial degree of toxicity or both. Of the many factors which limit their use, cellular uptake of nucleic acids and particularly subsequent intracellular trafficking to reach the target site are the most important [175]. Despite these existing obstacles there are currently an increasing number of promising candidates entering clinical trials (see Table 1). The pace observed over the last few years in developing alternative delivery strategies fosters hope that in the near future an arsenal of different nucleic acid-based drugs might become available to fight viral diseases.

**Table 1:** Antiviral oligomeric nucleic acids in clinical trials.

Drug	Class	Virus	Target transcript/s	Developer/Reference	Status
Fomivirsen (Vitravene^TM^)	asON	CMV	IE2	Isis Pharmaceuticals	Approved
VRX496	asON	HIV	*env*	[32]	Phase II
Morpholino asON	asON	ZEBOV and MARV	VP24 and VP35	[35,36]	Entering Phase I
OZ1	ribozyme	HIV	*tat* and *vpr*	[50]	Phase II
SPC3649	anti-miRNA	HCV	miR-122	Santaris Pharma	Phase I
ALN-RSV01	siRNA	RSV	N-protein transcript	Alnylam	Phase II
siRNA-SNALP	siRNA	ZEBOV	L-polymerase, VP24 and VP35	Tekmira Pharmaceuticals	Pre-clinical
